# RHAMM splice variants confer radiosensitivity in human breast cancer cell lines

**DOI:** 10.18632/oncotarget.7258

**Published:** 2016-02-08

**Authors:** Alexandra Schütze, Christian Vogeley, Tobias Gorges, Sören Twarock, Jonas Butschan, Anna Babayan, Diana Klein, Shirley K. Knauer, Eric Metzen, Volkmar Müller, Verena Jendrossek, Klaus Pantel, Karin Milde-Langosch, Jens W. Fischer, Katharina Röck

**Affiliations:** ^1^ Institut für Pharmakologie und Klinische Pharmakologie, Universitätsklinikum der Heinrich-Heine-Universität, Düsseldorf, Germany; ^2^ Department of Tumor Biology, University Medical Center Hamburg-Eppendorf, Hamburg, Germany; ^3^ Institute of Cell Biology (Cancer Research), University Hospital, University of Duisburg-Essen, Essen, Germany; ^4^ Institute for Molecular Biology II, Centre for Medical Biotechnology (ZMB), University of Duisburg-Essen, Essen, Germany; ^5^ Institute of Physiology, Faculty of Medicine, University Duisburg-Essen, Essen, Germany; ^6^ Department of Gynecology, University Hospital Hamburg-Eppendorf, Hamburg, Germany

**Keywords:** breast cancer, ionizing radiation, RHAMM, cell death, extracellular matrix

## Abstract

Biomarkers for prognosis in radiotherapy-treated breast cancer patients are urgently needed and important to stratify patients for adjuvant therapies. Recently, a role of the *receptor of hyaluronan-mediated motility* (*RHAMM*) has been suggested for tumor progression. Our aim was (i) to investigate the prognostic value of *RHAMM* in breast cancer and (ii) to unravel its potential function in the radiosusceptibility of breast cancer cells. We demonstrate that *RHAMM* mRNA expression in breast cancer biopsies is inversely correlated with tumor grade and overall survival. Radiosusceptibility *in vitro* was evaluated by sub-G1 analysis (apoptosis) and determination of the proliferation rate. The potential role of *RHAMM* was addressed by short interfering RNAs against *RHAMM* and its splice variants. High expression of *RHAMM*v1/v2 in *p53* wild type cells (MCF-7) induced cellular apoptosis in response to ionizing radiation. In comparison, in *p53* mutated cells (MDA-MB-231) *RHAMM*v1/v2 was expressed sparsely resulting in resistance towards irradiation induced apoptosis. Proliferation capacity was not altered by ionizing radiation in both cell lines. Importantly, pharmacological inhibition of the major ligand of *RHAMM*, hyaluronan, sensitized both cell lines towards radiation induced cell death. Based on the present data, we conclude that the detection of *RHAMM* splice variants in correlation with the *p53* mutation status could help to predict the susceptibility of breast cancer cells to radiotherapy. Additionally, our studies raise the possibility that the response to radiotherapy in selected cohorts may be improved by pharmaceutical strategies against RHAMM and its ligand hyaluronan.

## INTRODUCTION

Radiotherapy has become standard of care for most breast cancer cohorts [1]. Radiation has significantly reduced the risk of local recurrence and also improved overall survival [2]. However, cancer cells can acquire radioresistance with its complementary risk of increased mortality rates [3]. Furthermore, radiotherapy has been shown to increase the risk of cardiovascular diseases [4]. Hence, discovery of targets predicting the response to radiotherapy as well as agents that sensitize cancer cells to ionizing radiation with low side effects, is of great interest.

Various biomarkers have improved the identification of cancer patients who benefit from personalized therapeutic strategies. For example, in breast cancer (BC) progesterone or estrogen receptor positive BC subtypes are generally treated by endocrine ablation therapies in combination with chemotherapy and/or radiotherapy. However, all of those BC subtypes respond equally to radiation and biomarkers helping to define the radiation regime are urgently needed.

In particular, biomarkers for intrinsic or acquired resistance of tumor cells to radiotherapy remain elusive. Mechanisms causing resistance to radiotherapy are diverse and poorly characterized. Recent evidence suggests that aberrant apoptosis may contribute to this phenomenon [6].

p53, a nuclear phosphoprotein, is an important mediator of cell growth control and apoptosis [7]. It is activated by a variety of stress signals, e.g. irradiation, in order to eliminate damaged cells from the host [8]. Increased aggressiveness of cancer is associated with the loss of function of *p53* and chemo- and radioresistance have already been correlated with deleted or mutated p53 proteins [9]. Thus, accurate molecular evaluation of the *p53* status could be used to stratify patients, who might respond to additional therapies, such as radiotherapy, leading to an improved prognosis. Furthermore, identified mutations in the *p53* gene might provide a potential target for clinical intervention strategies. Theoretically, reversion to wild type p53 should restore cell growth control, apoptosis, or radiosensitivity, but has proven to be difficult to achieve [10]. Hence, the identification of downstream effectors of p53 could present novel therapeutic targets to reinforce radiosensitivity. However, the exact *p53* affected genes, responsible for radiation induced apoptosis, remain poorly characterized.

Recently, the receptor for hyaluronan-mediated motility (RHAMM) has been identified as a novel effector protein of p53 [11]. RHAMM acts as a cell-surface receptor for hyaluronan (HA) and as intracellular stabilizer of the mitotic spindle [12]. Its functional role is thought to be the response to pathological process and was shown to be increased in various tumors [13]. *RHAMM* is located on chromosome 5q33.2 and four different isoforms, generated by alternative splicing of its messenger RNA, have been described within the last years. Evidence exists that alternative splicing of *RHAMM* is involved in promoting formation of metastases of hepatic cancers [14]. As a consequence of its ability to bind HA, an extracellular matrix component known to promote tumorigenesis [14], RHAMM activates signaling pathways which have been implicated in BC progression [15] and cellular survival [16].

Aim of the present study was to investigate the functional role of RHAMM-proteins in BC as well as the relevance of its interaction with p53 with regard to therapeutic interventions supporting radiotherapy-based treatment decisions. In particular, the hypothesis was tested if RHAMM and its binding partner HA are eligible as therapeutic targets to sensitize breast cancer cells to ionizing radiation.

## RESULTS

### RHAMM is prognostic for overall survival in breast cancer patients and alters cancer cell phenotype in *in vitro* studies

**Figure 1:**
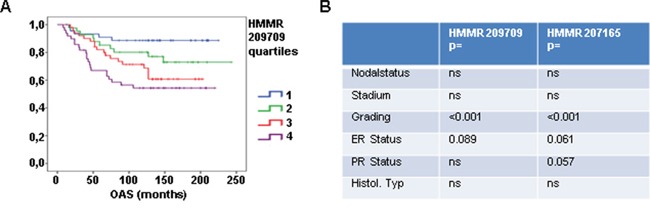
*RHAMM* is prognostic for patient overall survival **A.** Affymetrix analysis of *RHAMM* expression in 196 tissue samples from breast cancer patients is shown. Patients were stratified into subgroups according their *RHAMM* expression (low (1), medium (2), high (3), very high (4)) and the subgroups were correlated to overall survival. **B.** table showing results of statistic tests for clinical parameter in two affymetrix analysis.

To characterize the relevance of *RHAMM* expression in BC progression, mRNA expression data (Affymetrix) from 196 BC tissue samples were analyzed. Patients were stratified into quartiles according to their *RHAMM* expression for both HMMR probe sets present on the Affymetrix chips. The expression level was correlated to clinical and histological prognostic parameters and patient outcome. Increase in *RHAMM* expression was significantly correlated with a decrease in overall survival (OS) in both probe sets (Fig. 1A, data of the second probe set not shown) as well as recurrence-free survival (data not shown). Furthermore, a significant relationship between *RHAMM* and tumor grading was observed (Fig. 1B).

**Figure 2:**
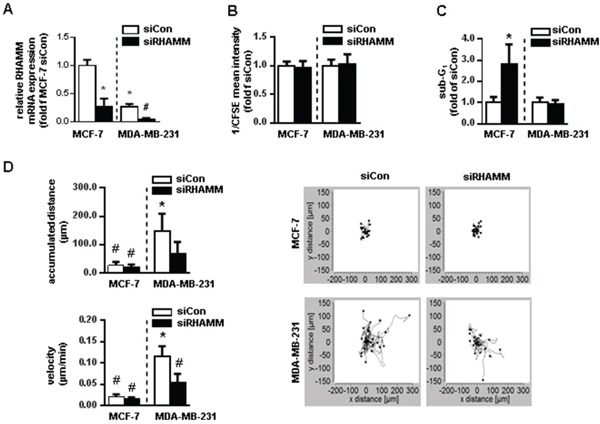
*RHAMM* has apoptotic and motility characteristics in different cancer cell lines *in vitro* **A.** relative mRNA expression of *RHAMM* in si*RHAMM* transfected MCF-7 and MDA-MB-231 cells. **B.** proliferation rate measured with CFSE staining in MCF-7 and MDA-MB-231 cells 48h after siRNA knockdown of *RHAMM*. CFSE intensity is assigned reciprocally. **C.** sub-G1 analysis of MCF-7 and MDA-MB-231 cells 48h after siRNA knockdown of *RHAMM*. **D.** accumulated distance and velocity of migration assay with exemplary pictures of one experiment. *, p<0.05 in comparison to MCF-7 siCon; ^#^, p<0.05 in comparison to MDA-MB-231 siCon.

Even though in previous studies RHAMM has been proposed as a prognostic marker in BC, its functional role remains largely unknown. Two different BC cell line cells (MCF-7 and MDA-MB-231) were used to test whether *RHAMM* influences cell proliferation, apoptosis, or migration. It has previously been described that cells of the MCF-7 line harbor high levels of RHAMM whereas cells of the MDA-MB-231 line express low levels of this protein [17, 18]. No effect on cellular proliferation quantified by CFSE and FACS analysis was observed 48h after transient inhibition of all RHAMM splice variants (Fig. 2A-2B). However, sub-G1 analysis revealed that si*RHAMM* treatment significantly increased the rate of cell death in MCF-7 cells whereas MDA-MB-231 cells were not affected (Fig. 2C). In contrast, knock down of *RHAMM* led to a decrease of migration in MDA-MB-231 cells whilst no change could be detected in MCF-7 cells (Fig. 2D).

### Ionizing radiation induces cell death in MCF-7 cells and leaves MDA-MB-231 cells unaffected

**Figure 3:**
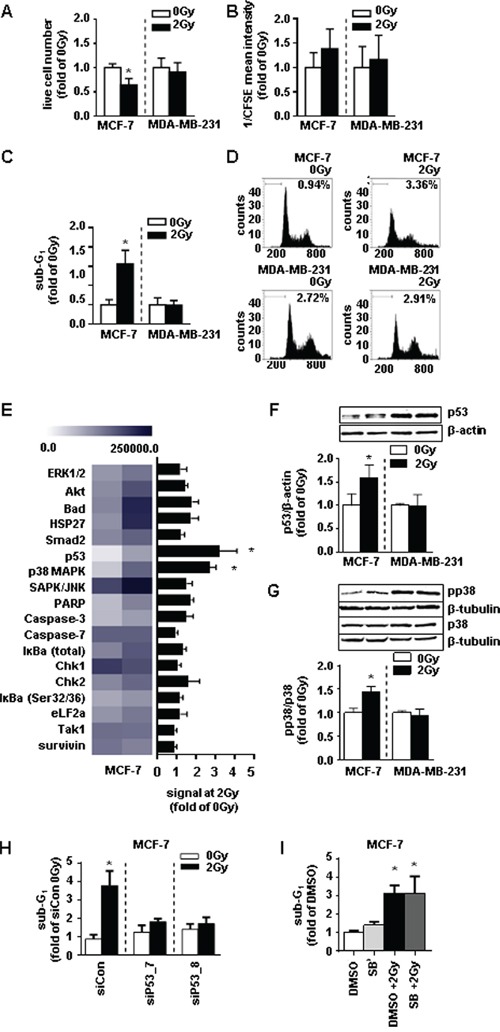
MDA-MB-231 cells are not radiosensitive at a dose of 2Gy whereas MCF-7 cells are radiosensitive via activation of p53 and p38 **A.** live cell number, **B.** proliferation rate of via CFSE (assigned reciprocally), and **C**. sub-G1 analysis of MCF-7 and MDA-MB-231 cells 48h after irradiation with 2Gy measurement. **D.** representative histograms of sub-G1 analyses. **E.** absolute expression levels of proteins analyzed via the stress and apoptosis assay depicted in a heatmap (left) and the fold changes of these proteins in MCF-7 cells 48h after irradiation compared to the non-irradiated control (right). **F.** western blot analyses of MCF-7 cell lysates for p53 and **G.** pp38 and p38 protein expression. **H.** sub-G1 analysis in MCF-7 cells transfected with siRNA against *p53* 48h after irradiation with 2Gy. **I.** proliferation rate of MCF-7 cells treated with SB (SB202190, p38-inhibitor, 10 μM) 4h before irradiation with 2Gy. *, p<0.05 in comparison with untreated MCF-7 0Gy (A-G, J, K)/siCon (H, I).

Since RHAMM appears to be implicated in tumor progression, the question was raised whether RHAMM has any implications in the cellular response to ionizing radiation. Initially, the susceptibility of both MCF-7 and MDA-MB-231, to 2Gy of ionizing radiation was characterized. As functional readout the rate of proliferation and cell death were chosen. The number of living cells was significantly reduced in MCF-7 cells after irradiation (Fig. 3A), whereas the proliferative capacity was not altered by 2Gy of ionizing radiation of both cell lines (Fig. 3B). MCF-7 cells revealed a significant increase in the apoptotic rate as measured by sub-G1 analysis explaining the decrease of the total cell number (Fig. 3C-3D). In comparison, MDA-MB-231 cells were found to be radioresistant (Fig. 3A-3D). In order to investigate the underlying mechanism of increased cellular death in MCF-7 cells, a protein array was performed analyzing the phosphorylation pattern of proteins involved in apoptosis. p53 and p38 were significantly increased in MCF-7 cells 48h after initial radiation (Fig. 3E-3G). This effect was not seen in MDA-MB-231 cells. Of note, MCF-7 cells harbor wild type p53 whereas MDA-MB-231 cells harbor a mutated form of the protein leading to its accumulation in the nucleus [19]. The involvement of p53 in the induction of apoptosis in MCF-7 cells was further validated by treatment with siRNA against *p53* prior to radiation. Short interfering RNA specific for *p53* abolished the pro-apoptotic effect of radiation in MCF-7 cells (Fig. 3H). Inhibition of p38 by SB202190 did not abrogate irradiation induced cell death. These results indicate that MCF-7 cell death in response to ionizing radiation is induced by the *p53* pathway.

Since both analyzed BC cell lines cells exhibit a divergent estrogen receptor (ER) status (MCF7:positive; MDA-MB-231 negative [18]), we next tested whether the ER is involved in the radiosusceptibility. However, treatment of MCF-7 cells with the unspecific ER-antagonist ICI182780 did not abrogate the pro-apoptotic effect of ionizing radiation in this cell type (Suppl. Fig. S1).

### RHAMM is regulated by radiation in a p53 correlated manner

**Figure 4:**
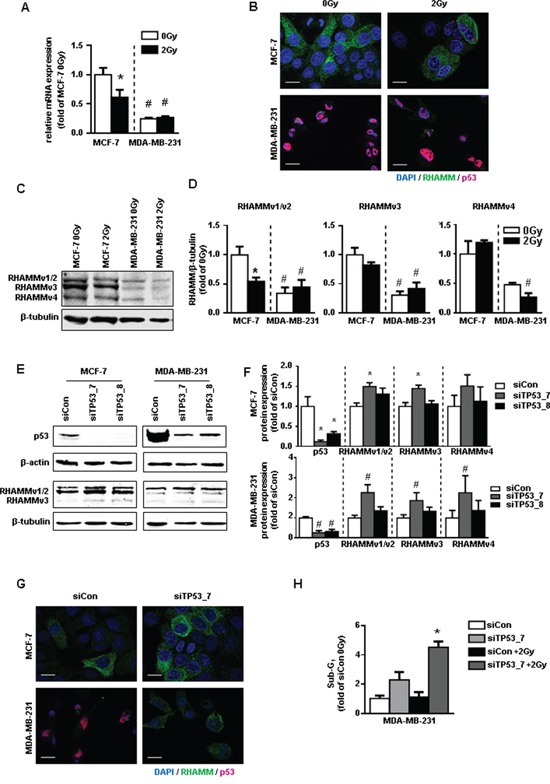
p53 and RHAMM variant status of MCF-7 and MDA-MB-231 in response to 2Gy irradiation **A.** relative mRNA expression of *RHAMM*pan. **B.** RHAMM (green) and p53 (pink) immunofluorescence staining 48h after irradiation with 2Gy. Scale bars: 20μm. **C.** western blot analysis of RHAMM protein expression and its quantification and **D.** quantification. **E.** exemplary blots of protein expression of p53 and RHAMM of *p53* depleted MCF-7 and MDA-MB-231 cells and **F.** quantification. **G.** RHAMM (green) and p53 (pink) immunofluorescence staining 48h after siRNA knockdown of p53. Scale bars: 20μm. H. MDA-MB-231 were transfected with sip53. 48h after transfection cells were irradiated. Sub-G1 was analyzed further 48h later.*, p<0.05 in comparison to MCF-7 siCon, #, p<0.05 MDA-MB-231 in comparison to MCF-7 siCon.

To investigate the role of RHAMM in response to radiation, both cell lines were irradiated with 2Gy and *RHAMM* mRNA level was measured by qRT-PCR (Fig. 4A). *RHAMM* mRNA was significantly reduced in MCF-7 cells, likewise shown by immunocytochemical staining of RHAMM (Fig. 4B). No change was detected in MDA-MB-231 cells resembling the results on the apoptotic response (Fig. 3C). Immunocytochemistry staining of p53 showed increased signals in irradiated MCF-7 cells compared to sham-irradiated controls. Accumulation of p53 in MDA-MB-231 cells was found independent of ionizing radiation (Fig. 4B). Alternative splicing of RHAMM might be responsible for different cellular functions. The regulation four different protein isoforms regulation of four by ionizing radiation was analyzed by western blot. Of note, only splice variants v1/v2 which both run at 85kDa were decreased in MCF-7 cells while v3 and v4 were not reduced. In contrast, MDA-MB-231 cells displayed a significantly lower expression of all tested isoforms and were not further decreased by radiation (Fig. 4C-4D). Recently, *RHAMM* has been shown to be transcriptionally repressed by p53 [11]. Treatment of both cell lines with short interfering RNA against *p53* confirmed these results (Fig. 4E-4G). The endogenously increased level of p53 in the nucleus of MDA-MB-231 cells could therefore explain the reduced occurrence of RHAMM protein in this cell line. Of note, reduction of RHAMM by siRNA did not change the level of p53 in both cell lines (Suppl. Fig. S2).

Next it was investigated whether the increase of RHAMM v1/v2 in MDA-MB-231 cells after p53 knock down would restore radiosensitivity. Knock down of p53 and subsequent upregulation of RHAMM v1/v2 increased the rate of cellular death in MDA-MB-231 cells. Of note, the apoptotic effect was even further enhanced by subsequent ionizing irradiation (Fig. 4H).

### RHAMM splice variants increase the radiosensitivity of breast cancer cell lines

**Figure 5:**
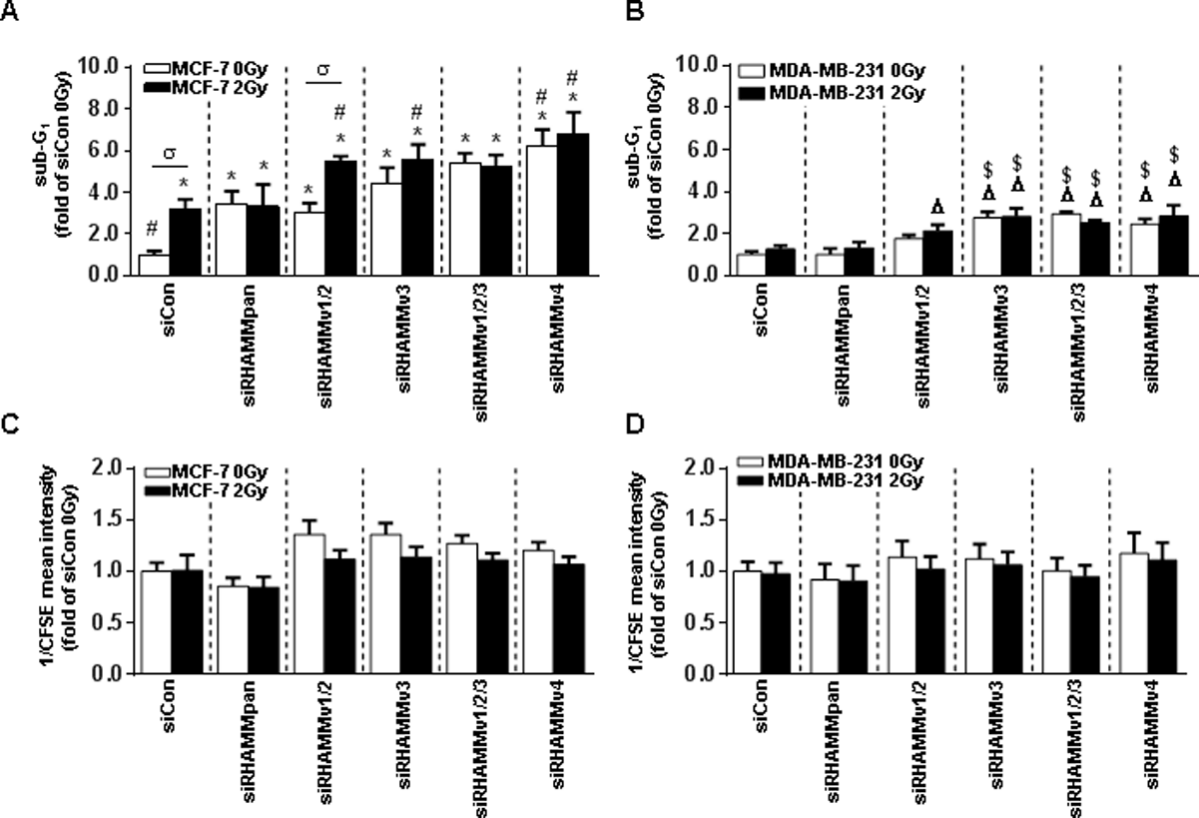
*RHAMM* pan and *RHAMM* variant knock-down in MCF-7 and MDA-MB-231 cells leads to increased radiosensitivity after irradiation with 2Gy **A.** sub-G1 cell cycle analysis of si*RHAMM* variants transfected MCF-7 and **B.** MDA-MB-231 cells 48h after irradiation with 2Gy. **C.** analysis of proliferation rate via CFSE measurement (assigned reciprocally) in si*RHAMM* variant transfected MCF-7 and **D.** MDA-MB-231 cells 48h after irradiation with 2Gy. *, p<0.05 in comparison to MCF-7 siCon 0Gy, ^#^, p<0.05 in comparison to MCF-7 siCon 2Gy, σ, p<0.05 MCF-7 siRHAMMv1/2 2Gy in comparison to MCF-7 siRHAMMv1/2 0Gy, Δ, p<0.05 in comparison to MDA-MB-231 siCon 0Gy, $, p<0.05 in comparison to MDA-MB-231 siCon 2Gy.

To establish the radiosensitizing ability of RHAMM - observed in terms of apoptosis - and to investigate the involvement of the different *RHAMM* splice variants, cells were treated with siRNA against the respective mRNAs (Suppl. Fig. S3) and subsequently irradiated. In MCF-7 cells si*RHAMM*pan as well as siRNA against all individual *RHAMM* splice variants increased the rate of apoptosis (white bars Fig. 5A).

Again, irradiation induced the percentage of apoptotic cells. An additional apoptotic effect was observed in cells which were treated with siRHAMM v1/v2 and irradiation. Knock down of RHAMM v3 and v4 did not alter this effect (Fig. 5A).

In MDA-MB-231 cells neither si*RHAMM*pan nor si*RHAMM*v1/v2 revealed a significant induction of cell death (Fig. 5B). However, siRNA against RHAMMv3 and v4 induced cell death in MDA-MB-231 cells. Irradiation did not increase MDA-MB-231 cell death in any treatment group.

As expected from the previous experiments, the proliferation rate was not altered by either ionizing radiation or siRNA treatment (Fig. 5C-5D).

### Pharmacologic inhibition of hyaluronan synthesis increases the response to ionizing radiation

**Figure 6:**
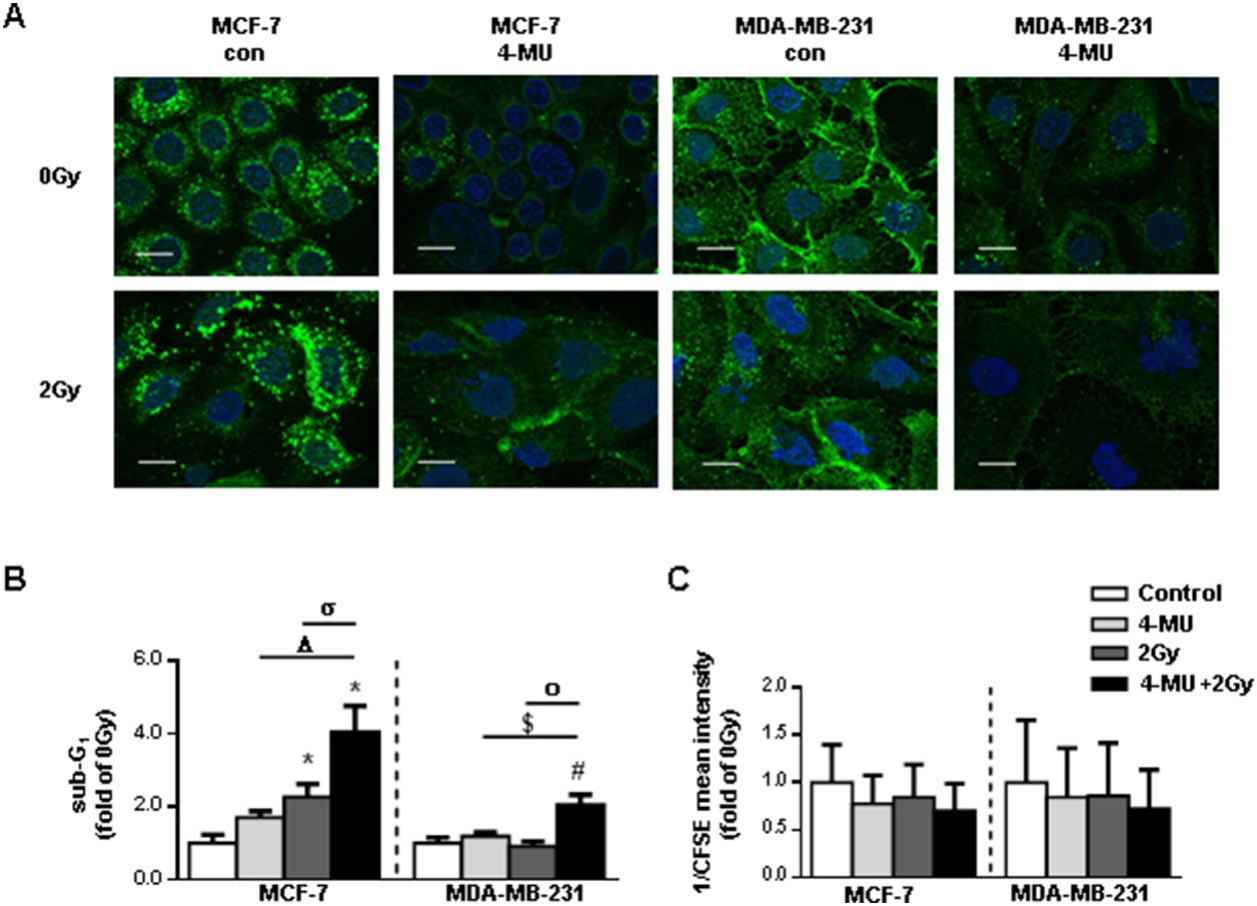
Pharmacological inhibition of HA system via 4-MU in MCF-7 and MDA-MB-231 leads to increased radiosensitivity **A.** affinitycytochemistry of HA 48h after irradiation with 2Gy. Scale bars: 20μm. **B.** sub-G1 cell cycle analysis of 4-MU treated MCF-7 and MDA-MB-231 cells 48h after irradiation with 2Gy. **C.** analysis of proliferation rate via CFSE staining of 4-MU treated MCF-7 and MDA-MB-231 cells 48h after irradiation with 2Gy. CFSE intensity is assigned reciprocally. *, p<0.05 in comparison to MCF-7 0Gy, Δ, p<0.05 in comparison to MCF-7 4-MU, σ, p<0.05 in comparison to MCF-7 2Gy, #, p<0.05 in comparison to MCF-7 0Gy, $, p<0.05 in comparison to MCF-7 4-MU, ο, p<0.05 in comparison to MCF-7 2Gy.

Finding pharmacological approaches which increase the susceptibility of cancer cells to radiation and thereby reduce the rate of recurrence is of great interest. HA is the main intra- and extracellular ligand of RHAMM thereby regulating both functional aspects [20]. Its pharmacological inhibition can be realized by treatment of cells with the HA-synthase inhibitor 4-methylumbelliferone (4-MU). 4-MU treatment suppressed HA synthesis in irradiated and non-irradiated breast cancer cells (Fig. 6A). Importantly, incubation of MCF-7 cells with 4-MU increased the radiosensitivity of the cells with respect to apoptosis fourfold. Whereas MDA-MB-231 cells did not respond to 4-MU treatment alone, the susceptibility of the cells after additional radiation was increased (Fig. 6B-6C).

**Figure 7:**
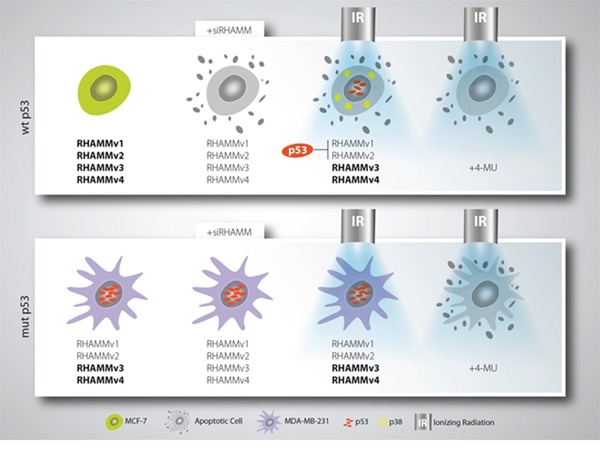
Schematic model of mamma-ca radiosensitization Breast cancer cells with wildtype *p53* status are radiosensitive and can be forced into apoptosis upon *RHAMM* downregulation. Mutant *p53* breast cancer cells are only radiosensitive if treated with 4-MU which affects RHAMM ligand inhibition.

In conclusion, we provide evidence, that RHAMM is involved in the malignant phenotype of BC cells. Detection of *RHAMM* isoform expression in correlation with the *p53* mutation status might allow to predict the responsiveness to radiation. Importantly, pharmacological inhibition of HA, the main binding partner of RHAMM, could help to increase the radiosensitivity of both *p53* wild type and mutated cancer types (Fig. 7).

## DISCUSSION

The receptor for hyaluronan-mediated motility (RHAMM) exhibits at least two distinct functions. As intracellular protein it is involved in maintaining the stability of the mitotic spindle [12]. In addition, by attachment to a GPI-anchor, it is associated with the cellular membrane and acts as cellular HA receptor promoting cell motility and invasion [15, 21]. Various studies report overexpression of RHAMM during tumor development and suggest a prognostic significance of RHAMM expression in e.g. leukemia, bladder cancer, and BC [13, 22, 23]. These findings have fostered the idea to use RHAMM for therapy in acute myeloid leukemia and multiple myeloma, now being evaluated as vaccination against RHAMM in small clinical trials [24]. However, its value as prognostic marker and therapeutic target remains indeterminate.

In agreement with other reports, our data show that the level of *RHAMM* in tumor biopsies derived from BC patients is correlated with recurrence-free and OS. Interestingly *in vitro* its functional role also appears to depend on the expression level. Whereas in RHAMM^high^ MCF-7 cells the cellular survival depends on the *RHAMM* expression, MDA-MB-231 cells (RHAMM^low^) survive independently of *RHAMM*. Of note, this circumstance is reversed with regard to the migratory capacity of both cell types. One possible explanation is that RHAMM is transported to the membrane in invasive cancer cell lines [21] and thereby increases migratory behavior. This suggests that (i) the amount of *RHAMM* mRNA is important for the prognosis of the disease and (ii) that the subcellular localization might be informative for the functional role. Future studies will have to be conducted to clarify this open issue.

Several factors may be associated with altered cellular radiosensitivity and the probability of cells to be killed by apoptotic mechanisms including *p53*, *FAS*-mediated pathways, and the *BCL-2* gene family [25]. The relationship between the level of endogenous apoptosis of tumors and their radiosensitivity has been investigated leading to conflicting results [26, 27]. However, it has been shown that cells from radiosensitive tumors are more susceptible to ionizing radiation compared to cells from unresponsive tumors [28]. Hence, the apoptotic response may serve as predictive assay for intrinsic radiosensitivity [29]. In the present study, a threefold increase in the apoptotic rate of RHAMM^high^ MCF-7 was detected. In comparison, RHAMM^low^ cells (MDA-MB-231) were found to be radioresistant. Apoptosis in caspase-3 deficient cells (MCF-7) has been reported to depend on alternative pathways like caspase-7 activation of DFF40 like DNAse [30]. In this study analysis of proteins involved in radiation induced cell death of MCF-7 cells revealed an involvement of p53 in this process. Of interest MDA-MB-231 cells harbor mutations in the *p53* gene [31], whereas MCF-7 express wild-type *p53* [32]. A number of tumors acquire *p53* mutations with a high frequency [33–36]. In general, patients with these mutations respond poorly to treatment with ionizing radiation.

In genome wide mRNA screens *RHAMM* was observed to be decreased when *p53* is active [7, 37, 38]. Furthermore, it was recently described that p53 can repress *RHAMM* expression via its promotor including the first exon and intron [11].

In the present study, we demonstrate that *RHAMM* expression in MDA-MB-231 was severely reduced in correlation with an accumulation of p53 within the nucleus. Even though it is generally postulated, that mutant *p53* cells have lost the ability to bind consensus *p53* DNA binding regions, it has been demonstrated that mutated *p53* is still able to regulate gene expression directly or through mitochondrial or cytoplasmatic activities [39]. In MDA-MB-231 cells it was previously reported that the p53 mutant is required for cellular survival through yet unknown mechanism [40]. In the present study siRNA against *p53* raised RHAMM protein levels indicating that mutated p53 in MDA-MB-231 cells is still able to repress *RHAMM* expression.

Of interest, not all *RHAMM* isoforms appear to be decreased by p53 as only *RHAMM*v1/v2 was reduced in MDA-MB-213 cells and elevated in response to siRNA against *p53*. Different functions of the *RHAMM* splice variants have been proposed previously. In multiple myeloma patients the ratio of *RHAMM*v3:*RHAMM*v1/v2 has been correlated with poor prognosis [16]. Furthermore *RHAMM*v3 appears to promote tumor growth and metastasis to lymph nodes and the liver in a mouse model [14]. As *RHAMM*v1/v2 encodes for longer proteins than *RHAMM*v3 and v4 and the transport mechanism of RHAMM to the membrane is still unknown, alternative splicing of *RHAMM* might present one reason for its presence in different subcellular compartments and thereby different cellular functions.

Regarding radiation responses the expression of RHAMM v1/v2 appears to be vital. Whereas RHAMMv1/v2^high^ MCF-7 cells were sensitive to ionizing radiation, RHAMMv1/v2^low^ MDA-MB-231 cells were radioresistant. It appears plausible that *RHAMM*v1/2 is permanently reduced in p53 mutated cancer cells and other compensatory pathways exist, thereby conferring radioresistance.

CD44 is a widely expressed cell surface membrane receptor which participates in cell-cell and cell-matrix interactions. CD44 facilitates mitogenic/invasive as well as proliferative cellular phenotypes [41]. Conflicting functions of CD44 have been proposed in experimental models of tumorigenesis and –progression in comparison to *in vivo* data. This may be due to the presence and absence of RHAMM and *vice versa* [42]. CD44 is known to co-operate with RHAMM and has been reported to compensate for loss of RHAMM. RHAMM and CD44 unify at least two distinct characteristics: i) both have been shown to be transcriptionally repressed by p53 [11, 43] and ii) they share the same binding partner, HA. HA is a glycosaminoglycan and an important component of the extracellular matrix which has been associated with BC progression [44]. *In vitro* HA induces BC cell motility [45] and survival [46]. HA has been predicted to bind all RHAMM isoforms near the carboxyl-terminus [47]. In the present study treatment with the HA-inhibitor 4-MU augmented the radiation effect in both cell lines. However, 4-MU did only alter MDA-MB-231 cellular survival in combination with irradiation. This hints towards a HA dependent resistance mechanism possibly mediated by CD44 or RHAMM v3/v4. Future studies will have to address the role of CD44 and RHAMM for radiosensitivity of cancer cells, possibly also including further binding partners of both proteins, such as MET [48]. Apart from 4-MU the application of other pharmacological compounds interfering with HA-signaling pathways, as RHAMM/CD44 blocking antibodies or HA-binding peptides, should be considered.

Prognostic markers may help to improve the accuracy of risk stratification of cancer patients and therefore possibly provide important data to optimize therapeutic decisions. In the present study we report that the detection of *RHAMM* splice variants in correlation with the *p53* mutation status might help to pre-evaluate the susceptibility of breast cancer cells towards radiotherapy. Additionally, the data raise the possibility that the response to radiotherapy in selected tumors may be improved by targeting RHAMM and its ligand HA.

## MATERIALS AND METHODS

### Analysis of mRNA expression data in tumor tissue samples

In order to investigate the mRNA expression of *RHAMM*, microarray data (Affymetrix HG-U133A) from a cohort of 196 mammary carcinoma enrolled in the Department of Gynecology, Hamburg University medical Center, were analyzed. The clinical and histological characteristics of this cohort as well as technical details have been described elsewhere [49]. All microarray data have been submitted to Gene Expression Omnibus (GEO) under the following accession numbers: GSE26971 (samples GSM663775-GSM663852), GSE31519 (samples GSM782523-GSM782529), GSE31519 (samples GSM782554-GSM782568), GSE46184 (samples GSM1125783-GSM112856) [49]. Informed consent for the scientific use of tissue materials, which was approved by the local ethics committee (Ethik-Kommission der Ärztekammer Hamburg, #OB/V/03), was obtained from all patients. The study was performed in accordance to the principles of the declaration of Helsinki and REMARK criteria [50].

Expression data of two RHAMM probe sets present on the Affymetrix chips (209709_s_at and 207165_at) were retrieved from the files. For statistical analysis, the cohort was divided into quartiles of similar size according to their expression values. χ^2^-tests were used to examine correlations between RHAMM expression and clinicopathological factors comparing the following groups: histological grading G1 vs. G2 vs. G3; stage pT1 vs. pT2 vs. pT3-4; lymph node involvement N0 vs. N1; estrogen and progesterone receptor status (ER, PR), positive vs. negative; histological type ductal vs. others. Overall survival was analyzed by Kaplan-Meier analysis and Log-Rank-Tests. Probability values less than 0.05 were regarded as statistically significant.

### Treatment and transfection of cells

MCF-7 (CLS, Eppelheim) and MDA-MB-231 (CLS, Eppelheim) were cultured in DMEM-high glucose (Gibco, Carlsbad) with 2% FBS (Gibco) and 1% Penicillin/Streptomycin (Gibco). Ionizing radiation was applied with Gulmay RS225 (Xstrahl, Camberley) at 150kV and 15mA with 0.2mm copper filter 24h after treatment or seeding of 5000 cells per cm^2^. 10nM siRNA (AllStars Negative Control siRNA, s*iHMMR*_9 (si*RHAMM*pan), si*TP53*_7 and si*TP53*_8 from Qiagen, Hilden and custom siRNA for *RHAMM* variants were purchased from Sigma) was used for transfection with 1:500 RNAiMax (Life Technologies, Carlsbad) in DMEM according to the manufacturer's manual. siRNA against sequences for knockdown of *RHAMM* variants are AAAGAGATTCGTGTTCTTCTACA (siRHAMMv1/2), AAGATTCGTGTTCTTCTACAGGA (siRHAMMv3), AAAGTTAAGTCTTCGGAATCAAA (siRHAMMv1/2/3), and AATGACCCTTCTGATTCGTGT (siRHAMMv4). Cells were irradiated 24h after reversed transfection with siRNA against *RHAMM* or 4-MU (Sigma-Aldrich Chemie, München) treatment. Cells receiving *p53* knockdown were irradiated 48h after reversed transfection with siRNA against *p53*.

### Migration assay

Migration of the cells was investigated via time lapse microscopy starting 24h after ionizing radiation with 2Gy. Three videos per sample were recorded with Axio Observer.Z1 under standard culturing conditions and eight cells per video were tracked. Tracking was performed using ImageJ 1.47t (Wayne Rasband, National Institutes of Health, USA) manual tracking plugin (Fabrice Cordeli, Institut Curie, Orsay, France) and exemplary pictures are processed with Chemotaxis and Migration Tool (Ibidi, Gerhard Trapp, Martinsried, Germany).

### Western blotting

Western blots were performed using standard procedures and the following reagents: RHAMM (GTX62573, GeneTex, Irvine), p53 (OP43, Calbiochem, Billerica), p38 (#9212, Cell Signaling Technologies, Danvers), pp38 (#9211, Cell Signaling Technologies, Danvers), β-tubulin (T7816, Sigma-Aldrich Chemie, München) and β-actin (A5316, Sigma-Aldrich Chemie, München). Binding protein for affinitycytochemistry of hyaluronan was biotinylated HABP (Calbiochem, Billerica) and as secondary antibody streptavidin-FITC (Dako, Glostrup) was used. Secondary antibodies for western blotting were IRDye^®^ 800CW goat anti-rabbit IgG, IRDye^®^ 800CW goat anti-mouse IgM, IRDye^®^ 680RD goat anti-rabbit IgG and IRDye^®^ 680LT goat anti-mouse IgM (LI-COR Biotechnology, Bad Homburg). Immunofluorescence secondary antibodies were AF488 goat anti-rabbit IgG (Life Technologies, Carsbad) and AF568 (Fab')_2_ fragment of goat anti-mouse IgG (H+L) (Life Technologies, Carsbad).

### Immunofluorescence staining and affinitycytochemistry of hyaluronic acid

For RHAMM and p53 immunofluorescent stainings cells were fixed with 4% paraformaldehyde (PFA). For hyaluronic acid affinitycytochemical staining cells were fixed with 70% EtOH/4% PFA/0.5% acidic acid. Stainings were performed as described previously [51, 52].

### RNA isolation, cDNA transcription, and qRT-PCR

RNA was isolation was performed according to the PeqLab peqGOLD TriFast (Erlangen) protocol. cDNA transcription was carried out with QuantiTect Reverse Transcriptase Kit (Qiagen, Hilden) according to the instruction manual. qRT-PCR was performed in duplicate on StepOnePlus RealTime PCR System using Platinum SYBR Green qPCR SuperMix-UDG (Life Technologies, Carlsbad) with ROX reference dye according to the manufacturer's protocol. Primer sequences (5′→3′) for *GAPDH* f are GTGAAGGTCGGAGTCAACG, GAPDH r TGAGGTCAATGAAGGGGTC, RHAMMpan f GAATTTGAGAATTCTAAGCTTG and *RHAMMpan* r CCATCATACCCCTCATCTTTGTT were used. Data were analyzed by ΔΔCT-method using *GAPDH* as reference gene.

### Intracellular signaling array

Cells were lysed in Path Scan^®^ Sandwich ELISA Lysis Buffer (Cell Signaling Technology, Danvers) and analyzed using PathScan^®^ Stress and Apoptosis Signaling Antibody Array Kit (Cell Signaling Technology, Danvers) according to the vendor's protocol. Chemiluminescent signals were detected via Odyssey Infrared Imaging System (application software version 3.0) from Li-Cor biosciences (Bad Homburg).

### Sub-G1 cell cycle analysis

Cells were detached via incubation withTrypsin/EDTA (Gibco), washed once with PBS and resuspended in 75μL Lysis buffer (0.1% Sodium citrate, 0.1% Triton X-100). Directly before FACS analysis 25μL GUAVA cell cycle reagent (Millipore, Billerica) were added and doublet-discrimination was performed via signal height/area dot plot. 10000 cells per sample were analyzed employing a guava easyCyte 5 flow cytometer (Millipore).

### Proliferation and determination of proliferation rate

Cells were detached and diluted 1:2 with TrypanBlue (Life Technologies, Carlsbad) for live dead discrimination and determination of live cell number in Countess (Invitrogen, Carlsbad).

To evaluate proliferation rate cells were stained with CFSE (Life Technologies, Carlsbad) as shown previously [53]. Cells were harvested 48h after irradiation. Analysis was performed employing the easyCyte5 flow cytometer (Millipore, Billerica). 10000 cells were analyzed per sample. Mean of fluorescence signal was assigned reciprocal.

### Statistical analysis

Real-time data were analyzed by logarithmic data transformation. Data were analyzed with GraphPad Prism 6 (GraphPad Software, La Jolla, CA, USA) and are represented as mean ± SEM. Comparison of two groups was analyzed via two-tailed t-test. Comparison of more than two independent variables was analyzed via one-way ANOVA and Sidak's multiple comparison *post hoc* test. Statistical significance was considered when p<0.05.

## SUPPLEMENTARY FIGURES


