# Supplementary material for: RHAMM splice variants confer radiosensitivity in human breast cancer cell lines
Source: Oncotarget. 2016 Feb 8;7(16):21428–40. doi: 10.18632/oncotarget.7258 (PMC5008296; doi:10.18632/oncotarget.7258)
Supplement: Supplementary file 1 [file oncotarget-07-21428-s001.pdf]

## SUPPLEMENTARY FIGURES

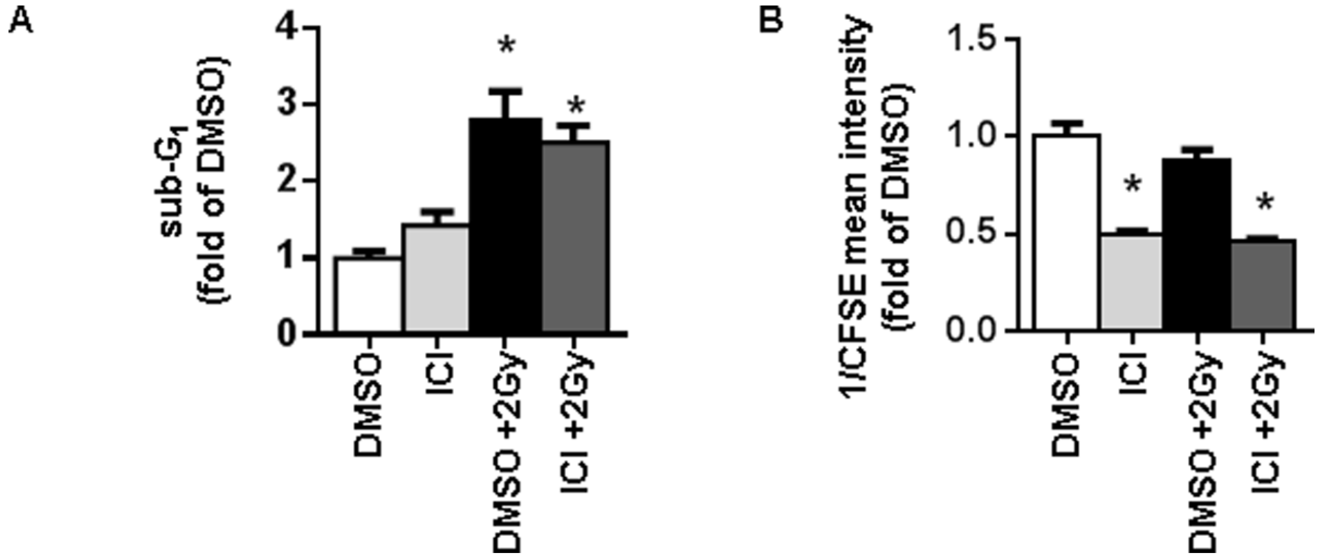


**Supplementary Figure S1: Estrogen receptor status has no effect on MCF-7 cell apoptosis but has an effect on proliferation. A.** sub-G1 analysis and **B.** proliferation rate of MCF-7 cells treated with ICI (unspecific ER-antagonist ICI182780, 10µM) 4h prior to irradiation and harvested 48h post irradiation with 2Gy. *, $p<0.05$ in comparison with MCF-7 cells treated with DMSO.

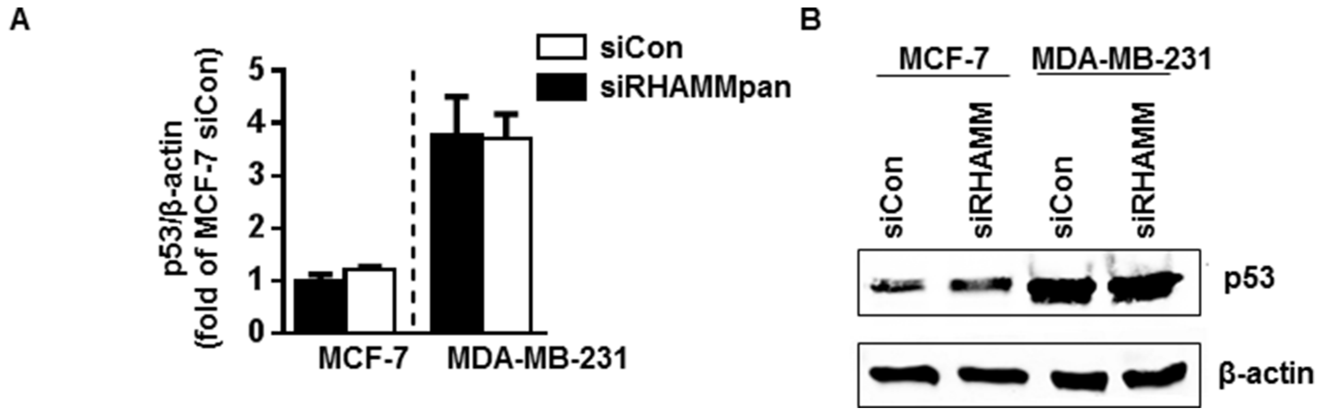


**Supplementary Figure S2: RHAMM knockdown has no effect on p53 expression. A.** western blot analysis of MCF-7 and MDA-MB-231 cells treated with siRNA against RHAMMpan 48h after transfection and **B.** representative blots.

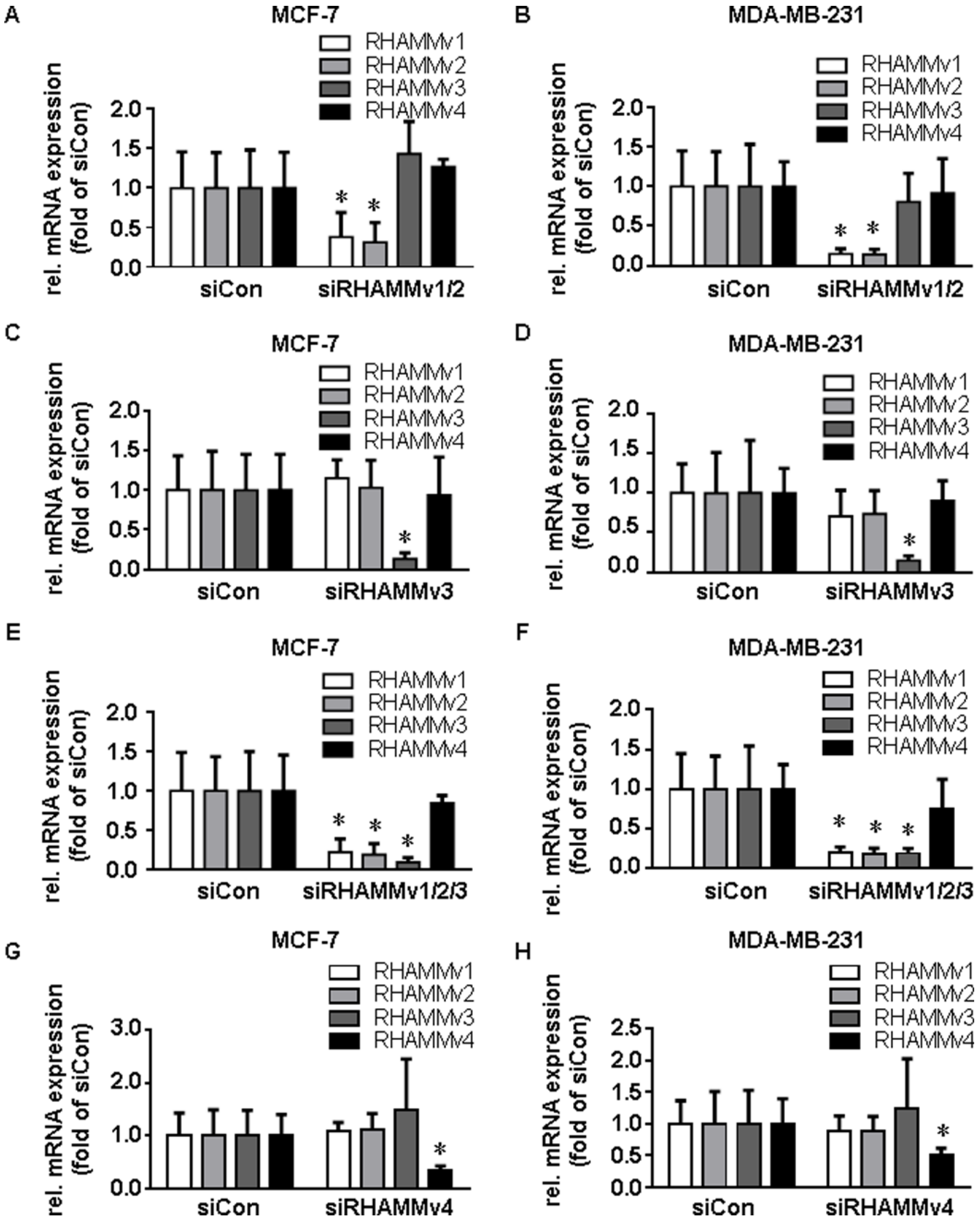


**Supplementary Figure S3: Knockdown efficacy of siRNA of *RHAMM* variants.** Relative mRNA expression of *RHAMM* variants in si*RHAMMv1/2* transfected **A.** MCF-7 and **B.** MDA-MB-231 cells. Relative mRNA expression of *RHAMM* variants in si*RHAMMv3* transfected **C.** MCF-7 and **D.** MDA-MB-231 cells. Relative mRNA expression of *RHAMM* variants in si*RHAMMv1/2/3* transfected **E.** MCF-7 and **F.** MDA-MB-231 cells. Relative mRNA expression of *RHAMM* variants in si*RHAMMv4* transfected **G.** MCF-7 and **H.** MDA-MB-231 cells. *, $p<0.05$ in comparison to respective siControl.
